# *TGFB1* and *LAMA1 gene* polymorphisms in children with high myopia

**DOI:** 10.12669/pjms.342.14616

**Published:** 2018

**Authors:** Elif Demirkilinc Biler, Orhan Ilim, Melis Palamar, Huseyin Onay, Onder Uretmen

**Affiliations:** 1 Elif Demirkilinc Biler, MD. Department of Ophthalmology, Ege University Faculty of Medicine, Izmir, Turkey; 2 Orhan Ilim, MD. Private Genesis Hospital, Diyarbakir, Turkey; 3 Melis Palamar, MD. Associate Professor, Department of Ophthalmology, Ege University Faculty of Medicine, Izmir, Turkey; 4 Huseyin Onay, MD. Associate Professor, Department of Ophthalmology, Ege University Faculty of Medicine, Izmir, Turkey; 5 Prof. Onder Uretmen, MD. Department of Ophthalmology, Ege University Faculty of Medicine, Izmir, Turkey.

**Keywords:** *TGFB1* gene polymorphism, *LAMA 1* gene polymorphism, High myopia

## Abstract

**Objective:**

To investigate *TGFB1* and *LAMA1* gene polymorphisms in children with high myopia in order to determine the genetic basis of large myopic shifts causing severe visual impairment and complications.

**Methods:**

Seventy-four children with high myopia (≥6 diopters [D]; study group) and 77 emmetropic children (±0.5D; control group) were included. Genetic and polymorphism analyses were performed in the Medical Genetics Laboratory using DNA purified from the patients’ blood samples.

**Results:**

Mean ages of the patients were 7.1±3 (3-13) and 9.6±1.8 (6-13) years in the study and control groups, respectively. Mean refraction in the high myopia group were -10.1±4.3D in the right and -8.9±3.6D in the left eye. *LAMA1* gene analysis of the study group revealed heterozygous mutations in 34 patients (45.9%), homozygous mutations in 25 patients (33.8%), and no mutations in the remaining 15 patients (20.3%). In the control group, there were 31 subjects (40.3%) with heterozygous, 27 (35.1%) with homozygous *LAMA1* mutations, and no mutations in 19 (24.7%) (p=0.73). *TGFB1* gene analysis showed heterozygous mutations in 32 (43.2%) and homozygous mutations in 10 patients (13.5%) in the study group, while 32 patients (43.2%) had no mutations. In the control group, 35 subjects (45.5%) had heterozygous, 8 (10.4%) had homozygous, and 34 (44.1%) had no *TGFB1* mutations (p=0.36).

**Conclusion:**

This is the first study to simultaneously examine two genes in high myopia in a Turkish population. However, we observed no significant differences in *TGFB1* and *LAMA1* gene polymorphisms in patients with high myopia compared to healthy subjects.

## INTRODUCTION

Myopia is an important refractive error that is common worldwide. High myopia is defined as refractive error of 6 Diopters (D) or more. Approximately 30% of myopic patients have high myopia.^1^,^2^ Myopic shifts of this degree can seriously impair vision and cause irreversible degenerative changes in the chorioretinal layer^3^ Retinal tears, retinal detachment, primary open-angle glaucoma, and visual field losses are common in these patients as myopia progresses with aging and the axial length of the eye continues to increase.^1^,^3^,^4^ Current treatments for high myopia still do not yield satisfactory results and there is no proven method of preventing high myopia.

Environmental and genetic factors are both known to play a role in the etiology of myopia, and high myopia is believed to be largely associated with genetic causes.^3^ To date, 341 genetic factors have been listed in the Online Mendelian Inheritance in Man (OMIM) database in relation to myopia. Genes implicated in the development of pathological myopathy include collagen type I (*COL1A1*), laminin alpha 1 (*LAMA1*), luminal (*LUM*), transforming growth factor beta 1 (*TGFB1*), paired box 6 (*PAX6*), insulin-like growth factor-1 (*IGF1*), zinc finger 644 (*ZNF644*), fibroblast growth factor 10 (*FGF10*), transforming growth factor beta-induced factor 1 (*TGIF1*), uromodulin-like 1 (*UMODL1*), and catenin delta-2 (CTNND2).^5^-^15^

Numerous studies have investigated the role of *TGFB1* and *LAMA1*, which are involved in the restructuring of scleral collagen and proteoglycans, in myopia. These studies were primarily conducted in Far East countries where the condition is more prevalent, and have revealed an association in certain populations.^5^,^6^,^16^-^21^

The *LAMA1* gene has received the most attention among those thought to be involved in high myopia. *LAMA1* is reportedly located on the short arm of chromosome 18 and forms the ~1648 kb centromere of the *ZFP161* gene in the MYP2 region.^22^ The gene encodes laminin, a structural glycoprotein component integral to the elastic system of the trabecular meshwork located in the scleral wall.^23^ In a recent study, *LAMA1* mRNA level was found to be lower in high myopic scleral tissue compared to that of non-myopic scleral tissue.^24^

Another gene implicated in the development of high myopia, *TGFB1*, is located at 19q13.1-q13.3’ in the human genome and contains seven exons.^25^ This gene encodes a multifunctional peptide that regulates proliferation, differentiation, migration, adhesion, and other functions in various cell types.^26^ *TGF-beta 1* is expressed in the sclera and can stimulate collagen production in scleral fibroblasts in a dose-dependent manner.^27^ Changes in *TGF-beta 1* expression were observed in animals during the development of experimental myopia.^28^ These findings suggest that *TGF-beta 1* may play an important role in the pathogenesis of myopia.

Understanding the role of genetic factors in nonsyndromic high myopia could enable the prevention of such extreme myopic refractive errors and associated complications, and direct future strategies for the management of this disease. In the present study we evaluated polymorphisms in the *LAMA1* (rs2089760) and *TGFB1* (rs4803455) genes in children younger than 13 years of age with <6 D myopia in an attempt to further elucidate the genetic basis of high myopia.

## METHODS

Seventy-four patients under the age of 13 years with high myopia (cycloplegic refraction values <6 D) and 77 emmetropic control children who presented to our Pediatric Ophthalmology Department were evaluated in the course of this study. Patients who had additional ocular pathology that may affect refraction (such as glaucoma, cataracts, corneal disease) and those with history of ocular surgery were excluded. All subjects underwent a complete ophthalmologic examination including strabismus examination, visual acuity assessment after correcting refractive error measured by autorefractometry, slit-lamp anterior segment examination, and 90-D lens posterior segment examination. A 2-cc blood sample was collected from each patient in EDTA-coated tubes and analyzed for *LAMA1* and *TGFB1* gene polymorphisms in the molecular genetics unit. The allele and genotype frequencies of the polymorphisms were compared between the study and control groups.

Data were analyzed in the SPSS 15.0 statistics program using the chi-square test and t-test. P value <0.05 was considered statistically significant. Informed consent forms were obtained from the legal guardians of all patients. The study was approved by a local ethics committee and conducted in accordance with the Declaration of Helsinki.

## RESULTS

The mean age of the patients with high myopia was 7.1±3 (3- 13) years; 38 were male and 36 were female. Their mean spherical refractive errors were -10.1±4.3 (-6.25 to -14.5) D on the right and -8.9±3.6 (-6.5 to -20.25) D on the left. The mean best corrected visual acuity of the 58 patients whose visual acuity level could be assessed was 5/10 (0.05-1.0) (Snellen) in both eyes.

The mean age of the patients in the control group (30 males, 47 females) was 9.6±1.8 (6-13) years. The control subjects had mean refractive errors of +0.22 D in the right and +0.26 D in the left eye, and all had uncorrected visual acuity of 10/10 (Snellen) in both eyes.

In the high myopia group (n=74), *LAMA1* gene analysis revealed heterozygous mutations in 34 patients (45.9%), homozygous mutations in 25 (33.8%), and no mutations in 15 patients (20.3%). In the control group (n=77), heterozygous *LAMA1* mutations were detected in 31 patients (40.3%), homozygous mutations in 27 (35.1%), and no mutations in 19 patients (24.7%). There was no statistically significant difference between the two groups in terms of *LAMA1* mutations (P=0.731, chi-square test) (Table-I).

**Table-I:** The distribution of study and control patients according to LAMA1 mutations.

LAMA 1	Study group (n=74)	Control group (n=77)
Wild type (no mutation)	15	19
Heterozygous mutation	34	31
Homozygous mutation	25	27

*TGFB1* gene analysis in the high myopia group revealed heterozygous mutations in 32 patients (43.2%), homozygous mutations in 10 (13.5%), and no mutations in 32 patients (43.2%). In the control group, 35 subjects (45.5%) had heterozygous *TGFB1* mutations, 8 (10.4%) had homozygous mutations, and no polymorphism was detected in 34 subjects (44.1%). There was no statistically significant difference between the two groups in terms of *TGFB1* mutations (P=0.358, chi-square test) (Table-II).

**Table-II:** The distribution of study and control patients according to TGFB1 mutations.

TGF B1	Study group (n=74)	Control group (n=77)
Wild type (no mutation)	32	34
Heterozygous mutation	32	35
Homozygous mutation	10	8

## DISCUSSION

The sclera forms the outermost layer of the eye and provides structural support to the globe. It is a connective tissue containing Extracellular Matrix (ECM) structures and fibroblasts. The sclera has a lamellar structure and significantly influences the dimensions of the eye. The collagen fibrils in the ECM are closely associated with proteoglycans and glycoproteins. Any change in ECM components is very likely to affect the shape and size of the globe. Dramatic changes in the ECM have been reported with growth/development or the progression of myopia.^4^ Significant pathologic changes occur in eyes with high myopia, the most common of which is scleral thinning in the posterior pole.

Laminin, a noncollagenous glycoprotein found in the ECM, consists of alpha, beta, and gamma chains, the latter of which are bound to the alpha chain. As a structural component of the scleral wall, laminin binds the microfibrils within and between the collagen fiber layers to the collagen fibrils in order to preserve scleral structure and function.^29^

Genetic factors play an important role in the development of pathological myopia. Although the functional studies conducted to date to investigate the implicated genes are limited in number and have reported more than a dozen candidate genes, most have been mapping or association studies. The pathogenic mechanisms of these genes at the transcription and translation levels remain unclear. While there is evidence that ECM changes and scleral remodeling play an important role in the development of high myopia, the specific pathogenic mechanisms have not been determined.^30^,^31^

In a study conducted in the Japanese population, Sasaki et al. compared 330 patients with >9 D myopia and 330 healthy volunteers and reported no significant association between *LAMA1* nucleotide sequence variants and high myopia.^16^ However, later studies supported the existence of such a relationship. Zhao et al. found that the rs2089760 polymorphism in the promoter region of the *LAMA1* gene was associated with high myopia in their study of 103 healthy subjects and 97 high-myopic patients in the Chinese population.^5^ Liang et al. also reported that the SNP rs2089760 G>A polymorphism in the promoter region of *LAMA1* was a significant factor in the development of myopia development.^17^ However, in our study there was no significant difference between the high myopia group and the control group in terms of *LAMA1* gene polymorphisms.

*TGF-beta 1* and its receptors are synthesized in ocular tissues and regulate collagen and matrix metalloproteinase production and fibroblast proliferation. There are numerous studies on the effect of *TGF-beta 1* on scleral remodeling during myopia development. *TGF-beta* and other growth factors have been shown to stimulate chondrocyte and fibroblast proliferation in cell cultures.^8^ Furthermore, a meta-analysis study revealed an association between *TGFB1* SNPs (rs1982073, rs4803455) and high myopia in Asians. Therefore, based on current evidence it was suggested that *TGFB1* may contribute to some degree to the development of myopia.^8^

In the present study, we observed no significant difference between the high myopia group and control group in terms of *TGFB1* gene polymorphisms. In a study comparing 288 patients and 288 healthy volunteers, Wang et al. reported no significant difference in the *TGFB1* gene.^18^ In contrast, *TGFB1* was reported to be the primary gene responsible for myopia in a study by Zha et al. including 300 high myopia (≥8 D) patients and 300 healthy subjects.^19^ Furthermore, a comprehensive meta-analysis by Meng et al. revealed a strong association between high myopia and *TGFB1* rs1982073 and rs4803455 SNPs.^22^ Other studies have also corroborated this finding.^6^,^21^

conclusion, studies conducted in different ethnic populations investigating the *TGFB1* and *LAMA1* genes, which are believed to be involved in high myopia, have yielded conflicting results. There is limited knowledge from our country and our population. Our study is the first to simultaneously examine two genes in a Turkish population; however, no significant polymorphisms were observed in high myopia patients when compared with healthy subjects. More detailed studies are needed to definitively establish the role of these genes in high myopia.

### Author’s Contribution

**EDB:** Conceived, designed, did the data collection, manuscript writing, statistical analysis and editing of manuscript.

**OI:** Did conception and design, data collection and manuscript writing.

**MP:** Did conception and design, analysis and interpretation of data and manuscript writing.

**HO:** Did conception and design, acquisition of data, analysis and interpretation of data.

**OU:** Did analysis and interpretation of data and editing of manuscript.

**EDB, MP &** **OU:** Takes the responsibility and is accountable for all aspects of the work in ensuring that questions related to the accuracy or integrity of any part of the work are appropriately investigated and resolved.
